# Antimicrobial resistance monitoring in the Danish swine production by phenotypic methods and metagenomics from 1999 to 2018

**DOI:** 10.2807/1560-7917.ES.2023.28.20.2200678

**Published:** 2023-05-18

**Authors:** Ana Sofia R. Duarte, Ana Rita Marques, Vibe D. Andersen, Helle B. Korsgaard, Hanne Mordhorst, Frederik D. Møller, Thomas N. Petersen, Håkan Vigre, Tine Hald, Frank M. Aarestrup

**Affiliations:** 1 Technical University of Denmark, National Food Institute, Kemitorvet 204, 2800 Kongens Lyngby, Denmark

**Keywords:** Surveillance, Antimicrobial resistance, metagenomics, correlation analyses

## Abstract

**Background:**

In Denmark, antimicrobial resistance (AMR) in pigs has been monitored since 1995 by phenotypic approaches using the same indicator bacteria. Emerging methodologies, such as metagenomics, may allow novel surveillance ways.

**Aim:**

This study aimed to assess the relevance of indicator bacteria (*Escherichia coli* and *Enterococcus faecalis*) for AMR surveillance in pigs, and the utility of metagenomics.

**Methods:**

We collated existing data on AMR and antimicrobial use (AMU) from the Danish surveillance programme and performed metagenomics sequencing on caecal samples that had been collected/stored through the programme during 1999–2004 and 2015–2018. We compared phenotypic and metagenomics results regarding AMR, and the correlation of both with AMU.

**Results:**

Via the relative abundance of AMR genes, metagenomics allowed to rank these genes as well as the AMRs they contributed to, by their level of occurrence. Across the two study periods, resistance to aminoglycosides, macrolides, tetracycline, and beta-lactams appeared prominent, while resistance to fosfomycin and quinolones appeared low. In 2015–2018 sulfonamide resistance shifted from a low occurrence category to an intermediate one. Resistance to glycopeptides consistently decreased during the entire study period. Outcomes of both phenotypic and metagenomics approaches appeared to positively correlate with AMU. Metagenomics further allowed to identify multiple time-lagged correlations between AMU and AMR, the most evident being that increased macrolide use in sow/piglets or fatteners led to increased macrolide resistance with a lag of 3–6 months.

**Conclusion:**

We validated the long-term usefulness of indicator bacteria and showed that metagenomics is a promising approach for AMR surveillance.

**Key public health message**

**What did you want to address in this study?**

Antimicrobial resistance (AMR) is a threat for treating infections, so it is surveillance is important. In pigs, AMR monitoring currently relies on phenotypic approaches. These consist in using indicator bacterial species, such as *Escherichia coli*, which are isolated from pig faeces and checked for their capacity to survive antimicrobials. We wanted to assess if metagenomics, a method for detecting and quantifying AMR genes, could also be useful for AMR surveillance.

**What have we learnt from this study?**

By comparing phenotypic and metagenomics results, we showed that employing *Escherichia coli* as an indicator for surveillance in the past 20 years has been appropriate. Like phenotypic surveillance, metagenomics allowed to describe changes in AMR in bacteria carried by Danish pigs over the study period (1999–2018). Changes in AMR during this period could be further compared or correlated with changes in the use of antimicrobials for treating animals.

**What are the implications of your findings for public health?**

Our study provides a validation step for possible future implementation of metagenomics for AMR surveillance. It also sets a potential foundation for further studies into the usefulness of metagenomics for other types of surveillance. Because metagenomics informs simultaneously on many AMR-contributing genes in faeces (regardless of the bacterial species these genes originate from) metagenomics could yield broader evidence for AMR monitoring.

## Introduction

Antimicrobial resistance (AMR) is a major global health threat. Multiple lines of evidence show that AMR bacteria emerge in livestock due to antimicrobial use (AMU), and that these AMR bacteria (or factors underlying their resistance capacity) might transmit from livestock to humans, including through food [1,2].

Surveillance is essential to follow trends of resistance over time and determine the effects of interventions such as reduction of AMU. In 1995, The Danish Integrated Antimicrobial Resistance Monitoring and Research Programme (DANMAP), at that time the world’s first continuous monitoring programme for AMR was established. It initially targeted livestock species and soon thereafter included human clinical and food samples [3,4]. Today DANMAP monitoring is based on a selected number of zoonotic and indicator bacterial species; it has functioned as a role model for similar integrated surveillance systems globally [5]. Long-term (i.e. ≥ 15 years) evaluation of the usefulness of selected indicator species has been only recently undertaken [6], and there is still no international consensus on a measure of AMR that summarises results of phenotypic resistance to multiple antimicrobial classes [7].

With recent developments in next-generation sequencing (NGS), it has become feasible to characterise the entire microbiome and resistome of any given sample, and in pig production, metagenomics analysis has shown promises for surveillance of AMR [8-10]. However, a long-term comparison between the widely used phenotypic approach and metagenomics has to our knowledge never been performed.

This study was conducted to compare the long-term value of the indicator bacteria, presently used in many AMR surveillance systems around the world, with metagenomics for surveillance of AMR. To do this we analysed the correlation between AMU and phenotypic resistance in indicator organisms and AMR resistance inferred from AMR gene (ARG) abundances during 1999–2018.

## Methods

### Antimicrobial use

DANMAP reports AMU based on Animal Daily Doses (ADDs). However, the reporting criteria changed in 2010 (www.danmap.org) to account for an increasing number of pigs being exported at the weight of 30 kg. Here we split the AMU data in two tranches: 2001–2009 and 2010–2018. For 2001–2009, we extracted AMU data per antimicrobial class directly from the DANMAP 2009 report (www.danmap.org) and AMU_2001–2009_ is expressed as total ADD/1,000 animals/year. For the period 2010–2018, we extracted total mg monthly use (*total amount of AMU*) of antimicrobial substances from the database VetStat (https://vetstat.fvst.dk/vetstat/), and subsequently calculated AMU_2010–2018_ as total ADD/kg-animals-at-risk/month, according to Formula [11]:

${AMU}_{2010-2018}=\frac{Total\;amount\;of\;AMU\;(mg)}{ADD\;\left(\frac{mg}{kg}\right)\times observation\;period\;\left(days\right)\times kg\;animals\;at\;risk}$

where *observation period* is the number of days of the period of the recorded AMU (in a given month) and *kg-animals-at-risk* is the biomass estimate as total kg-animals-at-risk in a given month, extracted from Danmarks Statistik (https://www.statistikbanken.dk/10472), for sows/piglets, weaners, or fatteners. All calculations were performed separately for each age category.

AMU_2010–2018_ was subsequently summarised at annual level, by adding the AMUs of all months of 1 year. Additionally, AMU of different substances of the same class were summarised under one antimicrobial class. The antimicrobial classes and substances present in both data tranches are indicated in supplementary table S1.

### Collection of caecal samples in the DANMAP programme

Details on the collection of caecal samples for the DANMAP programme are provided elsewhere [12,13] and great care has been taken to ensure comparable sampling over the years, accounting for the changes in the Danish swine production and slaughterhouse system. In brief, caecal samples from fatteners are collected monthly from all major slaughterhouses in Denmark. The number of samples per slaughterhouse is proportionally adjusted to the number of animals slaughtered per annum. The samples are collected in a systematic random manner by meat inspection or company staff, who are instructed to ensure that a farm is sampled only once. The caecal samples containing 30–100 g of caecal material from a single animal are submitted to the diagnostic laboratory for isolation of indicator *Escherichia coli* and *Enterococcus faecalis.* During the periods 1999–2004 and 2015–2018, the remaining individual caecal material which had not been used for *E. coli* or *E. faecalis* isolation had been frozen and subsequently stored at the Technical University of Denmark.

### Phenotypic resistance by broth microdilution

Monthly phenotypic resistance data for *E. coli* and *E. faecalis* isolated from caecal samples of individual fatteners were gathered for the period 2001–2018 from the DANMAP programme (www.danmap.org). An overview of available data are given in supplementary tables S3 and S4.

### Genotypic resistance by shotgun metagenomics

We had 67 pooled caecal samples available for shotgun-sequencing, with each pool corresponding to a mix of 0.1 g from each of 25 individual samples that were collected from fatteners at slaughter under the DANMAP programme throughout two time periods: 1999–2004 and 2015–2018. All individual samples in a given pool had been randomly selected among the available samples corresponding to a given month in a given year. Because 10 of the 67 pooled samples represented duplicates, we did not consider five of these 10. DNA was extracted from 0.2 g of each remaining 62 pooled sample. The 62 pooled caecal samples were shotgun sequenced as previously described [14,15]. An overview of the sequenced samples is given in supplementary table S4.

Raw FASTQ reads were quality- and adapter-trimmed using BBduk2, which is part of the BBmap suite of NGS tools [16]. We removed common adapters and trimmed the 3′-end using a Phred score of Q20, corresponding to a 1% error rate. The trimmed reads were mapped against the ARG database ResFinder [17] (downloaded 05 Sep 2019) and an internal genomic database including bacterial and other genomes (created 17 Oct 2019), and the relative abundance of individual ARGs was calculated as fragments per kilobase million (FPKM) as described previously [15]. Supplementary figure S1 shows the normalised counts of individual ARGs within each resistance class, by each sampling year.

As a multivariate, compositional dataset only relative changes are relevant, and thus analysis of compositions should be based on ratios or log-ratios [18,19]. Here we used centred log-ratios (clr) for ordination analysis and cluster visualisation and additive log-ratios (alr) for all multivariate statistical analyses. We transformed the normalised fragment counts to clr, according to equation:

$clr\left(x\right)=\left[{log}_{2}\frac{x_{1}}{g\left(x\right)}\ldots {log}_{2}\frac{x_{D}}{g\left(x\right)}\right]$

where *x* is the vector of normalised fragment counts for *D* ARGs in a sample and *g(x)* is the geometric mean of normalised fragment counts across *D* ARGs within a sample.

We transformed the normalised fragment counts to alr, according to equation:

$alr\left(x\right)=\left[{log}_{2}\frac{x_{1}}{y_{N}}\ldots {log}_{2}\frac{x_{D}}{y_{N}}\;\right]$

where *x* is the vector of normalised fragment counts for *D* ARGs in a sample, *and y_N_* is the total number of fragment counts for bacteria in the sample, i.e. alr corresponds to log_2_ (FPKM).

Transformations were performed with counts aggregated at (i) individual gene level, (ii) resistance class level and (iii) predicted resistance phenotypes (according to phenotypes annotated to individual genes in ResFinder). Normalised read counts were summed by class or phenotype before transformation.

### Heatmaps and ordination analysis

We visualised on heatmaps clr-transformed ARG counts, aggregated at antimicrobial class level, using the R package *pheatmap* v.1.0.12 [20], based on Euclidean distances and using Ward’s minimum variance agglomeration method without squared dissimilarities. Heatmaps were annotated by year and included clustering for antimicrobial class.

### Procrustes analysis

We performed Procrustes rotation analysis to assess the correlation between the annual AMU and phenotypic resistance in indicator *E. coli* and *E. faecalis*, and resistance determined by shotgun metagenomics sequencing. For *E. faecalis*, complete data were only available for 2001–2004. For the period 2015–2018, data were only available for 2 years, which was not enough to perform Procrustes analysis. Data were aggregated at antimicrobial class level, and analyses included subsets of the classes aminoglycoside, (am)phenicol, beta-lactam, macrolide, (fluoro)quinolone, sulfonamide, tetracycline and trimethoprim, according to the overlap between datasets.

First, we performed principal coordinates analysis (PCoA) for each of the multivariate datasets divided by sampling period, using the *cmdscale* function of R package *stats* [21] with a distance matrix based on Bray–Curtis distances estimated with the function *vegdist* of the package *vegan* v2.5–6 [22]. A Cailliez correction for negative eigen values was applied using the function *is.euclid* from the package *ade4* [23]. Next, we performed a symmetric Procrustes rotation analysis using the function *procrustes* of the *vegan* package to test the non-randomness (significance) between the configuration of two PCoAs. Then we tested the significance of the Procrustes statistic with the function *protest* of the same package, which uses a correlation-like statistic (*r*) derived from the symmetric Procrustes sum of squares (*ss*).

### Multivariate analysis of variance

A one-way multivariate analysis of variance (MANOVA) was performed to investigate significant changes within the period 2015–2018 in mean relative abundance (mean alr) of different resistance classes and predicted phenotypes. Data were split, before statistical analysis, into subsets by antimicrobial class and by predicted phenotype. Summary statistics were obtained with the function *get_summary_stats* of the R package *rstatix* v. 0.5.0. MANOVA was performed on a generalised multivariate linear model produced with the function *lm* of the package *stats* [21]. The function *Anova* of the package *car* [24] was then used to obtain MANOVA summary tables including: (i) Pillai-test statistic and its p-value for the multivariate change of the variables included in the linear model, (ii) F-test statistics and their p-values for the univariate change of the variables included in the linear model, p-values for the univariate F-tests corrected for simultaneous inference by term by the Holm method, and the sum of squares for sampling year and for residual error (SSE).

### Time-series cross-correlation analysis

We analysed the association between the monthly trends in AMU and AMR using metagenomic data aggregated at the class level, for the period 2015–2018, for which we had monthly data. A frequent and simple method to determine whether there is a relationship between two time series is via examination of their cross-correlation. A similar approach was used to determine cross-correlation of monthly AMU and AMR measured by phenotypic resistance in indicator *E. coli* in the same period. Details describing the analysis protocol and rationale are available in supplementary information.

## Results

### Antimicrobial use

**Figure 1:**
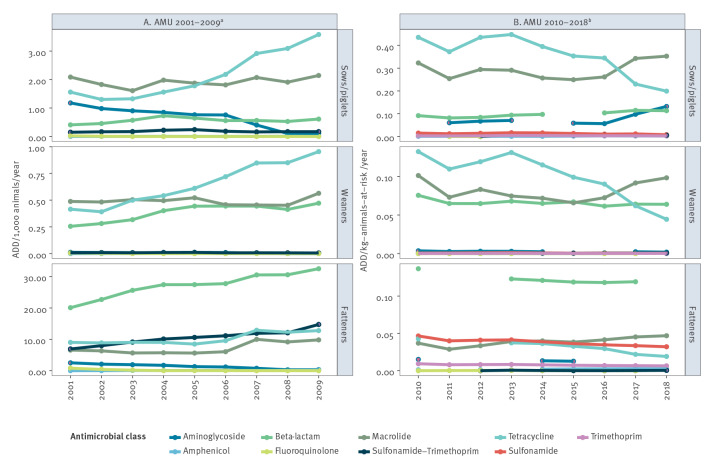
Annual antimicrobial use in sows/piglets, weaners and fatteners, Denmark, 2001–2018 (n = 18 years)

ADD: animal daily doses; AMU: antimicrobial use.

^a^ AMU was estimated as ADD/1,000 animals/year.

^b^ AMU was estimated as ADD/kg-animals-at-risk/year.

We obtained data on AMU for 16 antimicrobial classes for the years 2001–2018. Records on the use of the combinations beta-lactam–aminoglycoside and lincosamide–aminoglycoside were only available for 2001–2009 and were thus excluded. Monthly AMU data were only available for 2010–2018. Antimicrobial classes overlapping between the two reporting periods and for which there were corresponding results of broth microdilution and metagenomic sequencing were aminoglycoside, amphenicol, beta-lactam, fluoroquinolone, macrolide, tetracycline, sulfonamide and trimethoprim (see supplementary table S1). For these classes, we visualised annual AMU at the farm for the age categories sows/piglets, weaners and fatteners (Figure 1).

From 2010 to 2011, there was a shift in the trend of tetracycline and macrolide use due to the implementation of the Yellow Card initiative in 2010.

In the period 2001–2009, tetracycline use mostly increased in sows/piglets and weaners and also in fatteners after 2005; beta-lactam use in weaners and fatteners increased across the period; aminoglycoside use decreased in sows/piglets and fatteners; and the use of sulfonamide–trimethoprim in fatteners increased steadily.

From 2010–2011, there was a shift in the trend of tetracycline and macrolide use, from increasing to decreasing. This shift was due to the implementation by the Danish Veterinary and Food Authority (DVFA) of the Yellow Card initiative in 2010 (www.danmap.org), to reverse an increasing trend in AMU. As part of this initiative, each year, the DVFA issues threshold limits for antimicrobial consumption at the farm level in three age groups of pigs. Prescriptions are monitored continuously online. If the limits are exceeded the farm is contacted and visited; the DFVA conducts follow-up visits if compliance is not subsequently achieved. Tetracycline use continued to decrease in all age classes after 2013, while macrolide use increased in all ages after 2016 and aminoglycoside use increased in sows/piglets after 2016.

In both sampling periods, the antimicrobial classes with overall highest use across all ages were beta-lactam, tetracycline and macrolide. Additionally, aminoglycoside use was high in sows/piglets, and sulfonamide use was high in fatteners.

### Phenotypic resistance

**Figure 2:**
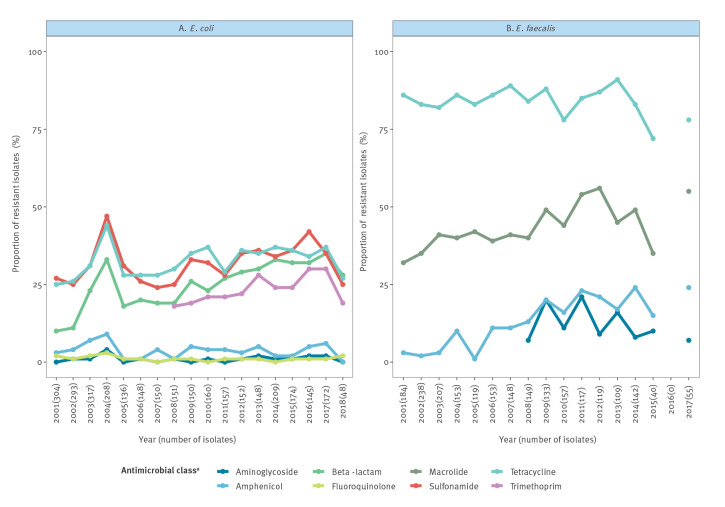
Annual proportion of resistant isolates of (A) *Escherichia coli* and (B) *Enterococcus faecalis*, determined by broth microdilution, Denmark, 2001–2018 (n = 3,222 *E. coli* and 2,223 *E. faecalis* isolates)

Data on antimicrobial susceptibility testing were available for 3,222 indicator *E. coli* isolates collected between 2001 and 2018, and 2,223 indicator *E. faecalis* isolates collected in 2001–2015 and in 2017. The estimated annual proportion of resistant isolates for each microorganism is illustrated in Figure 2. An overview of the antimicrobial substances included in the minimum inhibitory concentration (MIC) panels in the different years is given in supplementary tables S2 and S3.

Annual percentage of (A) *E. coli* and (B) *E. faecalis* isolates phenotypically resistant to an antimicrobial class, as determined by broth microdilution (y-axis). The number of isolates tested in a year is given in parentheses on the x-axis. Inside the graph in panel B, the representations of the different distributions appear interrupted at the level of the year 2016, because there were no data for *E. faecalis* in pigs for 2016.

^a^ Antimicrobial classes are represented by selected individual antimicrobial substances: aminoglycoside (gentamicin), amphenicol (chloramphenicol), beta-lactam (ampicillin), fluoroquinolone (ciprofloxacin), macrolide (erythromycin), sulfonamide (sulfamethoxazole), tetracycline (tetracycline).

Among *E. coli* isolates, the respective proportions of isolates resistant to sulfonamides, tetracycline, beta-lactams and trimethoprim were consistently higher than those resistant to amphenicols, aminoglycosides and fluoroquinolones, whereas for *E. faecalis* resistance was most common for tetracycline followed by macrolides (Figure 2). In *E. coli,* we observed a general increase over time in phenotypic resistance to tetracycline, sulfonamide, trimethoprim and beta-lactams. In *E. faecalis*, we observed an overall decrease in resistance to tetracycline and an overall increase in resistance to macrolides and amphenicol.

### Resistome

**Figure 3:**
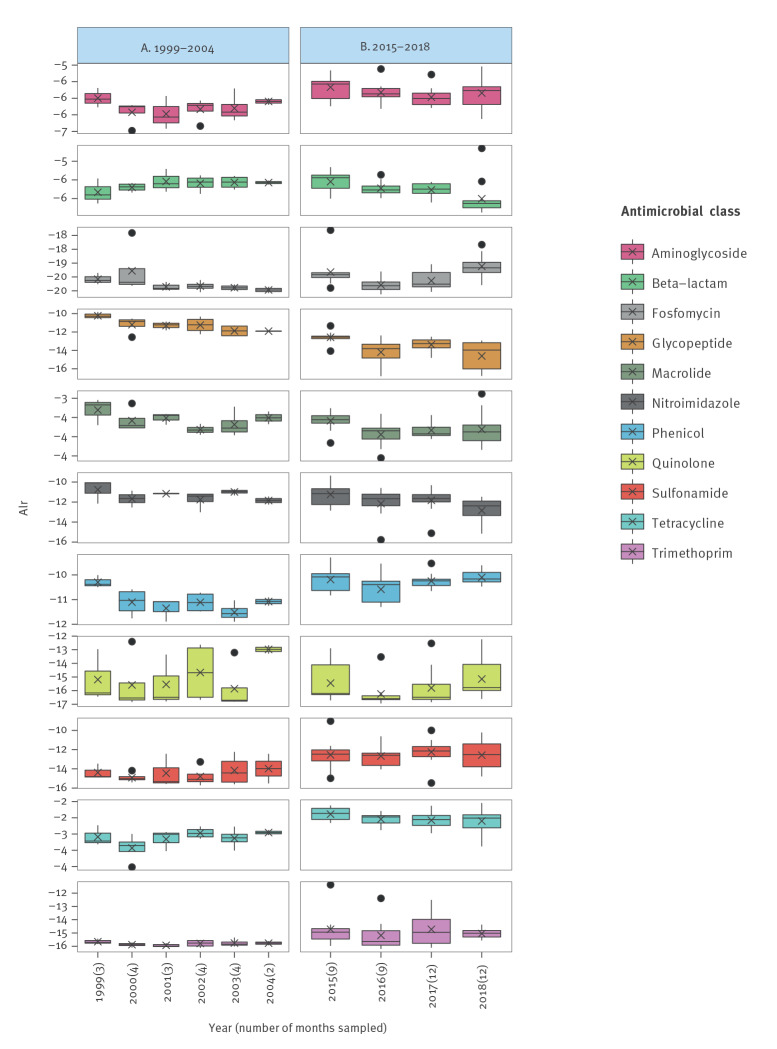
Relative abundance of antimicrobial resistance classes in pig resistomes in (A) 1999–2004 and (B) 2015–2018, Denmark, 1999–2018 (n = 62 pooled faecal samples)

We obtained metagenomic (resistomes) data for a total of 62 pooled faecal samples collected from pigs at slaughter (supplementary table S4). A total of 272 individual ARGs were identified. Relative abundance of resistance was inspected with data aggregated at ARG level (supplementary figure S1) and at antimicrobial class level (Figure 3).

Alr: additive log-ratios; ARG: antimicrobial resistance gene; IQR: interquartile range.

Relative abundance of antimicrobial class resistance (alr-transformed ARG counts, aggregated by antimicrobial resistance class) with variation between samples collected within a year. The boxplots represent the variation of the relative abundance of an antimicrobial class between the months sampled within a given year (i.e. the distribution of alr values): each box contains values between the 25^th^ percentile (Q1) and the 75^th^ percentile (Q3) IQR, with the horizontal line representing the median; the vertical line below the box starts at the minimum value (Q1 − 1.5 × IQR), the vertical line above the box ends at the maximum value (Q3 + 1.5 × IQR), and the dots below and above represent outliers, below the minimum or above the maximum values, respectively. The cross symbol represents the mean relative abundance of resistance to each class within a year. On the x-axis, the number of months sampled is given in parentheses.

In 1999–2004, the relative abundance of beta-lactam- and sulfonamide resistance increased, while the relative abundance of resistance to glycopeptide, macrolide and phenicol overall decreased, in the first case in a steady manner, and in the two latter in an oscillating manner.

In 2015–2018, we observed an overall decrease in the relative abundance of resistance to beta-lactam, nitroimidazole, glycopeptide and tetracycline, and, mostly from 2016, an overall increase in the abundance of resistance to fosfomycin, phenicol and trimethoprim, (Figure 3). Relative abundance of aminoglycoside, tetracycline, sulfonamide, trimethoprim and phenicol resistance was seemingly higher in 2015–2018 compared with 2001–2004, while relative abundance of glycopeptide resistance was clearly lower. 

During both periods, relative abundance of resistance was highest for aminoglycoside, beta-lactam, macrolide and tetracycline and lowest for fosfomycin, trimethoprim and quinolone.

When visually comparing the values of percentages of phenotypically-resistant *E. coli* isolates with the values of relative resistance in the resistome (supplementary figure S2), we observed a general agreement between both approaches in that values for tetracycline and beta-lactam resistance were higher than those for fluoroquinolone resistance. However, while values for sulfonamides and trimethoprim resistance were high among phenotypically-resistant *E. coli*, they were low in the resistome. Concerning resistance to aminoglycosides, on the other hand, the opposite was observed. In general, resistance was relatively constant over time for the resistome measurement, whereas proportions of phenotypically-resistant isolates among the *E. coli* seemed to fluctuate more.

### Clustering of resistomes suggested changes in AMR over time

The relative abundance of the ARG counts formed two main clusters agglomerating most samples from latest years (2016–2018) separately from those of earliest years (1999–2004), with samples from year 2015 spread among both clusters (supplementary figure S3).

**Figure 4:**
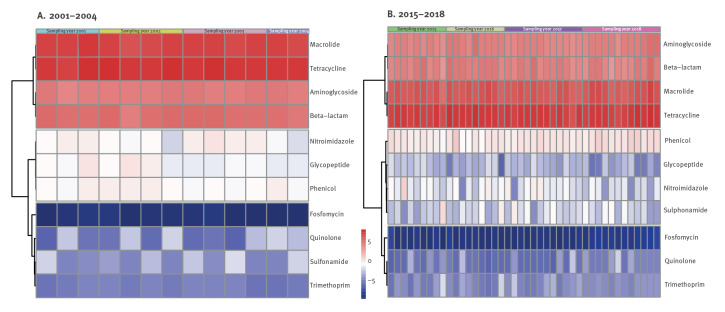
Clustering of resistomes aggregated at antimicrobial resistance class level, Denmark, 2001–2004^a^ and 2015–2018 (n = 62 pooled faecal samples)

Individual ARGs formed another two clusters, separating genes with overall low relative abundance over time, from genes with intermediate and high relative abundance. We observed no evident clustering according to antimicrobial class with gene-level counts. However, with counts aggregated at class level, antimicrobial classes clustered based on their general relative abundance across sampling years (Figure 4). In both sampling periods, two main clusters separated the four most relatively abundant classes (macrolide, tetracycline, beta-lactam and aminoglycoside) from least abundant ones. Each main cluster was further sub-divided based on general gradients of relative abundance, i.e. classes with lowest relative abundance over time and classes with intermediate relative abundance.

Clr: centred log ratio (clr).

^a^ Because heatmaps at antimicrobial resistance class level were produced to further enable comparisons between resistome and antimicrobial use, the start of the first period for CLR calculations was 2001, corresponding to the first year of the current study when data on antimicrobial use were available.

Clr transformed count matrices were used to produce a heatmap at antimicrobial resistance class level for (a) sampling period 2001–2004 and (b) sampling period 2015–2018. The value for each class in a sample represents that class’s ratio to the average abundance across all classes in the same sample. Samples were clustered by row (antimicrobial class) based on Euclidean distances and by Ward agglomeration method (dissimilarities not squared) and were annotated by year. The continuous colour scale represents clr values, from red (high) to blue (low).

In 2001–2004, classes with the lowest abundance were fosfomycin, quinolone, sulfonamide and trimethoprim, while glycopeptide, phenicol and nitroimidazole presented intermediate relative abundance. In 2015–2018, sulfonamide resistance shifted to the cluster of intermediate relative abundance. 

Among the four classes with highest abundance over time, macrolide and tetracycline were the most abundant in both periods. While quinolone- and glycopeptide resistance showed obvious lower relative abundance in the second sampling period, phenicol-, sulfonamide- and trimethoprim resistance showed obvious higher relative abundance. These observations were in accordance with the results presented by sampling year (Figure 3 and supplementary figure S2).

### Correlations of AMU, phenotypic resistance and resistome

**Figure 5:**
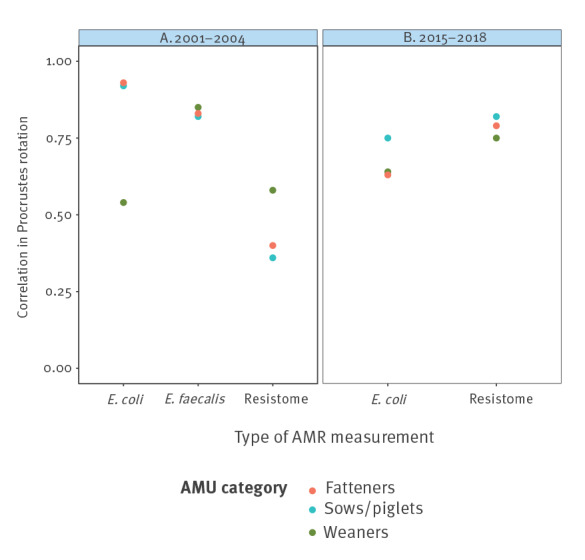
Correlation between AMU in three age categories and phenotypic- and genotypic AMR measured in fatteners at slaughter, estimated by Procrustes rotation analysis, Denmark, 2001–2004 and 2015–2018 (n = 1,661 *Escherichia coli* isolates, 782 *Enterococcus faecalis* isolates, 62 pooled faecal samples)

AMU: antimicrobial use; AMR: antimicrobial resistance.

^a^ AMR is assessed in *E. coli* and *E. faecalis* isolates with phenotypic methods as well as in samples through genotypic methods (metagenomics) which give the resistome.

Correlation between genotypic resistance and AMU appeared consistently high (*r* value > 0.7) for the period 2015–2018 (*r* values of 0.82 for sows/piglets, 0.75 for weaners and 0.79 for fatteners) (Figure 5 and supplementary table S5). In 2001–2004, that correlation seemed noticeably lower across all age groups (Figure 5). The correlation between phenotypic resistance in indicator *E. coli* and AMU in 2015–2018 appeared to be comparable to, but lower than, the correlations observed with genotypic resistance (*r* values of 0.75 for sows/piglets, 0.64 for weaners and 0.63 for fatteners). The correlation between phenotypic resistance in indicator *E. faecalis* and AMU in 2001–2004 seemed high for all age categories (*r* values of 0.82 for sows/piglets, 0.85 for weaners and 0.83 for fatteners). In terms of resistance trends according to AMU correlations between genotypic and phenotypically resistant *E. coli* or *E. faecalis* appeared low (*r* value ≤ 0.50) in both periods (supplementary table S5). It should be noted that except for tetracycline use in fatteners and tetracycline resistance in *E. coli* none of the correlations mentioned above were significant at the 5% significance level.

### Significant changes in predicted resistance phenotypes

We used MANOVA to identify significant changes in the resistome composition between the two sampling periods with metagenomics data available (1999–2004 and 2015–2018) for both predicted AMR phenotypes, and individual ARGs (supplementary table S6). All AMR classes annotated to a single phenotype showed a significant difference between the two periods, with sulfonamide- and trimethoprim resistance being significantly higher in the latest period, and glycopeptide significantly lower (supplementary table S6).

Some classes presented significant changes in opposite directions among their annotated phenotypes. For example, the overall significant change in aminoglycoside resistance was due to higher gentamicin-, streptomycin- and tobramycin relative abundance in 2015–2018, concomitantly with a lower amikacin relative abundance (supplementary figure S4a). Other classes, such as macrolide–lincosamide–streptogramin (MLS), showed both positive and negative significant changes, as well as nonsignificant changes among all annotated phenotypes, (supplementary figure S4b).

An additional MANOVA further allowed to identify which specific ARGs significantly contributed to the estimated change in a given phenotype (see supplementary table S6).

### Increase in AMU led to significant increase in relative abundance in resistome

We analysed time-lagged cross-correlations between AMU and AMR measured by shotgun-metagenomics and by phenotypic resistance in indicator *E. coli*. The cross-correlation coefficients are given in supplementary tables S7 and S8, respectively. Multiple significant correlations were observed but we focused our attention on positive coefficients with negative lags, which indicate increased AMU leading to increased AMR (supplementary figure S5). Here we observed that in multiple cases increased AMU led to increased genotypic AMR measured at slaughter, with a time-lag of between 3 and 11 months, except for the use of amphenicol in weaners, which was significant for a lag of 16 months (supplementary table S7). For *E. coli* increased AMU was only found significantly correlated with changes in sulfonamide and tetracycline resistance, and only with a time-lag of more than 1 year.

Due to the very large number of significant and contradictory correlations observed, we expect a high false discovery rate in these findings. However, even though further studies are clearly needed, these preliminary results suggest that changes in AMU may more easily be detected in the resistome.

## Discussion

Here we determined the multivariate correlation between AMU and AMR as measured by metagenomics and by phenotypic resistance in two commonly used indicator bacteria, *E. coli* and *E. faecalis*. AMU expressed in ADD/kg-animals-at-risk showed high, but non-significant, correlation, with phenotypic resistance in *E. coli* and with genotypic resistance; slightly higher with the latter. Results were less comparable for the first sampling period, where AMU was expressed in ADD/1,000 animals. In this period AMU had a higher correlation with phenotypic resistance in *E. coli* and *E. faecalis* than with resistance measured by metagenomics. It has been previously shown that results of AMU in pigs differ based on reported units [25] and that interpretation of surveillance data can be highly influenced by the AMU indicator used, for example due to the biomass denominator applied [26]. Our data, however, do suggest that ADD/kg-animals-at-risk is a preferable measurement of AMU, especially when considering the integration of metagenomics in AMR monitoring.

The cluster-analysis of resistomes showed in general a consistent separation between antimicrobial classes with overall lowest relative abundance, from those with overall highest relative abundance. Furthermore, it was possible to visually identify antimicrobial classes with increased or decreased resistance in the second sampling period compared with the first, with increase in sulfonamide resistance as the most obvious example represented by a shift in cluster between periods. Previous efforts have been made for monitoring approaches to integrate results of phenotypic AMR to different antimicrobial classes into a common indicator [7]. The cluster analysis of metagenomics results is an obvious asset to support the development and validation of such a single indicator.

We also identified antimicrobial classes with a statistically significant change in relative gene abundance between the periods 2001–2004 and 2015–2018, with genes of most classes, showing an overall significant increase in relative abundance during the second period. Moreover, within each antimicrobial class, we could predict individual phenotypes from the ARGs, as well as which specific ARGs contributed to the overall significant change. It is thus possible to determine significant changes in the abundance of AMR genetic determinants at the desired level of classification – from individual resistance gene to (predicted) resistance phenotype or general AMR class. This is one of the advantages of applying metagenomics in AMR monitoring, as opposed to phenotypic testing, where a limited set of bacteria–resistance phenotype combinations are used, and often a single combination is considered representative of a whole antimicrobial class. As our results show, within one class, the trend of individual phenotypes might be contradictory. This level of detailed analysis could eventually help unravel the effect on AMR of subtle changes in AMU practices and support the adaptation of new interventions at an earlier stage. Furthermore, it allows a timely detection of the emergence in a reservoir of ARGs associated with specific phenotypes. A MANOVA of resistomes can be a comprehensive method for the selection of target genetic determinants for a subsequent trend analysis, such as the one performed recently on commensal *E. coli* monitoring data [6].

Interestingly we observed a continued presence but constant decreasing abundance of glycopeptide resistance, despite the complete stop of any use of glycopeptides in the Danish livestock production back in 1995 [27]. This also shows that it will take very long time to reduce selected AMR even after complete bans on antimicrobial use.

We also investigated the association between monthly trends in AMU and AMR, in the period 2015–2018. For this type of association, significant cross-correlation coefficients were in general more frequent and higher in value with genotypic AMR than with phenotypic AMR. The only result consistent between the two measures of resistance was the increased use of sulfonamide in sows/piglets leading to an increase in sulfonamide resistance in finishers at slaughter (supplementary tables S5 and S8; supplementary figure S5). Interestingly, increased AMU in weaners did not lead to significant increase in indicator *E. coli* resistance at slaughter for any of the individual antimicrobial classes. Also, association between tetracycline use in fatteners and tetracycline resistance was only observed with phenotypic resistance but not with resistome. For several significant associations, lags of more than 10 months between increase in AMU and increase in AMR were observed. While a lag of 10 months or more may seem unreasonable at the individual farm level, it has been previously shown that AMU and AMR can be associated considering a lag of more than 1 year with national monitoring data [28]. Conclusions from these results are limited by the fact that different combinations of antimicrobial classes/substances were available for the cross-correlation analysis with phenotypic- and genotypic AMR. However, they indicate that AMR monitoring with metagenomics allows the identification of statistical association between trends in AMU and occurrence of AMR.

The current study is despite compiling data over 18 years still limited by the representativeness in the number of samples and years investigated. In addition, it is difficult to compare the data obtained from the indicator bacteria, which only constitute a very minor part of the bacterial community in the samples, with the metagenomics data. Furthermore, it is difficult to link the observed ARGs to a specific bacterial species, even though metagenome assembled genomes (MAGs) might in the future aid overcoming this challenge [29]. Nonetheless this study clearly shows that metagenomics can describe the AMR changes over time in entire livestock populations and that resistome changes can be compared with changes in AMU, with the additional advantage that metagenomics data can be shared under open access for future (re)use.

The European harmonised monitoring of AMR in bacteria from animals and food is adapting towards the reporting and analysis of genomics data, and the most recent legislation allows for the voluntary reporting of whole genome sequencing (WGS) results for selected bacteria [30]. It is thus expected that most European countries will soon have acquired the necessary infrastructure and skills to establish WGS-based AMR surveillance, which will possibly pave the way for the implementation of metagenomics. The transmission of antimicrobial resistance from animals to humans through for example the food chain implies some degree of contamination and potential transfer of ARGs from commensal bacterial species in the food chain or the human gut [31,32]. Thus, in the future surveillance of not only single bacterial species but the entire resistome might provide a better quantification of food borne risks compared to the current surveillance. 

### Conclusion

Our results suggest that the hitherto use of *E. coli* as indicator organism for AMR in surveillance has been appropriate for the purpose of following AMR trends and relating them to changes in AMU. However, we envisage that a similar or even improved level of AMR monitoring might be achieved with metagenomics in the future, since this approach offers deeper knowledge about the pool of ARGs existing in a reservoir. Thus, with further validation, metagenomics may become a valid option for AMR monitoring, as a complement to or potential future replacement for phenotypic testing.

## Supplementary Data

891000 Supplement
