# Supplementary material for: Antimicrobial resistance monitoring in the Danish swine production by phenotypic methods and metagenomics from 1999 to 2018
Source: Euro Surveill. 2023 May 18;28(20):2200678. doi: 10.2807/1560-7917.ES.2023.28.20.2200678 (PMC10197494; doi:10.2807/1560-7917.ES.2023.28.20.2200678)
Supplement: Supplement [file 22-00678_AARESTRUP_supplement.pdf]

Supplementary data, results and method description for the article [Antimicrobial resistance monitoring in the Danish swine production by phenotypic methods and metagenomics from 1999 to 2018]

Disclaimer: This supplementary material is hosted by *Eurosurveillance* as supporting information alongside the article [Antimicrobial resistance monitoring in the Danish swine production by phenotypic methods and metagenomics from 1999 to 2018], on behalf of the authors, who remain responsible for the accuracy and appropriateness of the content. The same standards for ethics, copyright, attributions and permissions as for the article apply. Supplements are not edited by *Eurosurveillance* and the journal is not responsible for the maintenance of any links or email addresses provided therein.

Antimicrobial use data

Supplementary table S1– Overview of antimicrobial use data available in the study for periods 2001-2009 and 2010-2018.

Antimicrobial class/substance	2001-2009 (year)	2010-2018 (year)	2010-2018 (month)
* Aminoglycoside	x	x	-
Apramycin	-	-	x
Gentamicin	-	-	x
Kanamycin	-	-	x
Neomycin	-	-	x
Spectinomycin	-	-	x
Streptomycin	-	-	x
*# Amphenicol	x	x	x
* Beta-lactam	x	x	x
*¥ Fluoroquinolone	x	x	x
* Macrolide	x	x	x
* Sulfonamide	-	x	x
* Trimethoprim	-	x	x
* Tetracycline	x	x	x
Sulfonamide-Trimethoprim	x	x	x
Beta-lactam - Aminoglycoside	x	-	-
Lincosamide - Aminoglycoside	x	-	-

*antimicrobial classes for which data for phenotypic- and genotypic- resistance were available (#amphenicol resistance measured with broth microdilution and phenicol resistance measured by metagenomics; ¥fluoroquinolone resistance measured with broth microdilution and quinolone resistance measured by metagenomics); *(year)* indicates data aggregated by year and *(month)* data aggregated by month.

Phenotypic resistance data

Supplementary table S2 - Overview of AMR substances for which *Escherichia coli* broth microdilution results were available in each sampling year within the study period.

AMR class	AMR substance	2001-2007	2008-2013	2014-2018
Aminoglycoside	Gentamicin*	x	x	x
	Apramycin	x	x	-
	Neomycin	x	x	-
	Spectinomycin	x	x	-
	Streptomycin	x	x	-
Beta-lactam	Ampicilin*	x	x	x
	Cefalotin#	x	-	-
	Cefotaxime	-	x	x
	Cefpodoxime ¤	x	-	-
	Ceftiofur	x	x	-
	Ceftazidime	-	-	x
Amphenicol	Chloramphenicol*	x	x	x
	Florfenicol	x	x	-
Fluoroquinolone	Ciprofloxacin	x	x	x
Sulfonamide	Sulfamethoxazole	x	x	x
Trimethoprim	Trimethoprim	-	x	x
Tetracycline	Tetracycline	x	x	x

* Substance chosen to represent an antimicrobial class, due to data completeness; # data not available for year 2001; ¤ data only available for years 2005-2007.

Supplementary table S3 - Overview of AMR substances for which *Enterococcus faecalis* broth microdilution results were available in each sampling year within the study period.

AMR class	AMR substance	2001-2013	2014	2015	2017
Aminoglycoside	Gentamicin[*¤]	x	x	x	x
	Kanamycin	x	-	-	-
	Streptomycin	x	-	-	-
Amphenicol	Chloramphenicol[*]	x	x	x	x
	Florfenicol[#]	x	-	-	-
Streptogramin	Quinopristin-Dalfopristin	x	x	x	x
Macrolide	Erythromycin	x	x	x	x
Tetracycline	Tetracycline	x	x	x	x

[*] Substance chosen to represent an antimicrobial class, due to data completeness; [#] data only available for years 2001-2007; [¤] data not available for years 2001-2007.

Genotypic resistance by shotgun metagenomics

Supplementary table S4 – sampling month and year of shotgun-sequenced samples available in the study

Sample ID	Year	Month	Total fragments	Total bacteria fragments
pig_1999_may_june_1_p_R1	1999	may	36595166	5750558
pig_1999_oct_2_p_R1	1999	oct	35658134	4767697
pig_1999_nov_3_p_R1	1999	nov	36234589	6316510
pig_2000_feb_4_p_R1	2000	feb	36949569	5767885
pig_2000_mar_5_p_R1	2000	mar	37076919	7582894
pig_2000_may_6_p_R1	2000	may	35097491	6582062
pig_2000_oct_7_p_R1	2000	oct	37948137	6273923
pig_2001_jan_8_p_R1	2001	jan	39676314	5963892
pig_2001_feb_9_p_R1	2001	feb	36803468	7022920
pig_2001_aug_10_p_R1	2001	aug	39598972	7339988
pig_2002_jan_11_p_R1	2002	jan	36094307	5692379
pig_2002_feb_12_p_R1	2002	feb	36524183	6555911
pig_2002_aug_13_p_R1	2002	aug	35985094	6762108
pig_2002_sept_14_p_R1	2002	sep	42094706	7708010
pig_2003_jan_15_p_R1	2003	jan	36368892	6694542
pig_2003_feb_16_p_R1	2003	feb	36327169	7153386
pig_2003_aug_17_p_R1	2003	aug	35979901	7434635
pig_2003_sep_18_p_R1	2003	sep	36157376	6364271
pig_2004_jan_19_p_R1	2004	jan	37461643	7903895
pig_2004_aug_20_p_R1	2004	aug	37283599	6736357
pig_2015_mar_21_p_R1	2015	mar	40698059	6948328
pig_2015_apr_22_p_R1	2015	apr	36470443	4899020
pig_2015_maj_23_p_R1	2015	may	36130801	5180195
pig_2015_juni_24_p_R1	2015	jun	36591745	4642571
pig_2015_juli_25_p_R1	2015	jul	37739513	4975702
pig_2015_aug_26_p_R1	2015	aug	38712662	4736955
pig_2015_sep_27_p_R1	2015	sep	36530284	6206788
pig_2015_oct_28_p_R1	2015	oct	36135053	5351272
pig_2015_nov_29_p_R1	2015	nov	38128057	4858530
pig_2016_jan_30_p_R1	2016	jan	39321507	5982573

pig_2016_feb_31_p_R1	2016	feb	36552251	7212620
pig_2016_apr_32_p_R1	2016	apr	36664368	5512367
pig_2016_maj_33_p_R1	2016	may	45473618	6692353
pig_2016_juni_34_p_R1	2016	jun	35701714	4601159
pig_2016_sept_35_p_R1	2016	sep	41647172	6561528
pig_2016_oct_36_p_R1	2016	oct	44558649	6348978
pig_2016_nov_37_p_R1	2016	nov	47380518	8143237
pig_2016_dec_38_p_R1	2016	dec	47156998	7658537
pig_2017_jan_39_p_R1	2017	jan	42017975	7641784
pig_2017_feb_40_p_R1	2017	feb	40123405	6591768
pig_2017_mar_41_p_R1	2017	mar	43172103	6766186
pig_2017_apr_42_p_R1	2017	apr	42910224	6582571
pig_2017_maj_43_p_R1	2017	may	38593345	6742847
pig_2017_juni_44_p_R1	2017	jun	38139931	5854981
pig_2017_juli_45_p_R1	2017	jul	35125980	4242661
pig_2017_aug_46_p_R1	2017	aug	33584083	4571300
pig_2017_sept_47_p_re_1_R1[¥]	2017	sep	30703220	3834542
pig_2017_oct_48_p_R1	2017	oct	27906287	4762473
pig_2017_nov_49_p_R1	2017	nov	37776957	6045369
pig_2017_dec_50_p_R1	2017	dec	45258204	7538233
pig_2018_jan_51_p_R1	2018	jan	37036078	3922534
pig_2018_feb_52_p_R1	2018	feb	30337186	4265352
pig_2018_mar_53_p_R1	2018	mar	31930154	4804033
pig_2018_apr_54_p_R1	2018	apr	36638350	4207900
pig_2018_maj_55_p_R1	2018	may	40815032	4694369
pig_2018_juni_56_p_R1	2018	jun	27527693	3873349
pig_2018_july_57_p_R1	2018	jul	31185099	5274764
pig_2018_aug_58_p_R1	2018	aug	29783836	3676254
pig_2018_sept_59_p_R1	2018	sep	29434116	4177710
pig_2018_oct_60_p_R1	2018	oct	28454603	2763211
pig_2018_nov_61_p_R1	2018	nov	27580212	3578543
pig_2018_dec_62_p_R1	2018	dec	27418504	6467973

Total fragments corresponds to the total number of fragments sequenced from each sample. Total bacteria fragments corresponds to the total number of fragments mapped to bacteria. [¥]sample has been re-sequenced due to low number of sequenced fragments in first round;

Procrustes analysis

Supplementary table S5 – Multivariate correlation of annual estimates of antimicrobial antimicrobial use with phenotypic resistance in indicators *Escherichia coli* and *Enterococcus faecalis*, and with genotypic resistance by metagenomics, by Procrustes rotation analysis.

Age class	Period	AM classes	Procrustes *ss*	Correlation *r*	Significance
**AMU vs. genotypic resistance**					
Sows/piglets	2001 - 2004	Aminoglycoside<br>Amphenicol<br>Beta-lactam<br>Macrolide<br>Tetracycline	0.87	0.36	0.88
Sows/piglets	2015 - 2018	Amphenicol<br>Beta-lactam<br>Macrolide<br>Tetracycline<br>Sulfonamide<br>Trimethoprim	0.33	0.82	0.21
Weaners	2001 - 2004	Aminoglycoside<br>Amphenicol<br>Macrolide<br>Tetracycline	0.67	0.58	0.67
Weaners	2015 - 2018	Aminoglycoside<br>Amphenicol<br>Beta-lactam<br>Macrolide<br>Tetracycline<br>Sulfonamide<br>Trimethoprim	0.44	0.75	0.13
Fatteners	2001 - 2004	Macrolide<br>Tetracycline	0.84	0.40	0.83
Fatteners	2015 - 2018	Amphenicol<br>Beta-lactam<br>Macrolide<br>Tetracycline<br>Sulfonamide	0.37	0.79	0.13

		Trimethoprim			
**AMU vs. phenotypic resistance in *E. coli***					
Sows/piglets	2001 - 2004	Aminoglycoside<br>Amphenicol<br>Beta-lactam<br>Tetracycline	0.15	0.92	0.13
Sows/piglets	2015 - 2018	Amphenicol<br>Beta-lactam<br>Tetracycline<br>Sulfonamide<br>Trimethoprim	0.43	0.75	0.29
Weaners	2001 - 2004	Aminoglycoside<br>Amphenicol<br>Tetracycline	0.76	0.49	0.54
Weaners	2015 - 2018	Aminoglycoside<br>Amphenicol<br>Beta-lactam<br>Tetracycline<br>Sulfonamide<br>Trimethoprim	0.59	0.64	0.46
Fatteners	2001-2004	Tetracycline	0.13	0.93	0.04*
Fatteners	2015 - 2018	Amphenicol<br>Beta-lactam<br>Tetracycline<br>Sulfonamide<br>Trimethoprim	0.60	0.63	0.42
**AMU vs. phenotypic resistance in *E. faecalis***					
Sows/piglets	2001 - 2004	Amphenicol<br>Macrolide<br>Tetracycline	0.32	0.82	0.13
Weaners	2001 - 2004	Amphenicol<br>Macrolide<br>Tetracycline	0.28	0.85	0.29
Fatteners	2001 - 2004	Macrolide<br>Tetracycline	0.31	0.83	0.08

Genotypic resistance vs. phenotypic resistance in *E. coli*				
**Period**	**AM classes**	**Procrustes SS**	**Correlation *r***	**Significance**
2001 – 2004	Aminoglycoside<br>Amphenicol<br>Beta-lactam<br>Fluoroquinolone<br>Tetracycline<br>Sulfonamide<br>Trimethoprim	0.83	0.41	0.83
2015 – 2018	Aminoglycoside<br>Amphenicol<br>Beta-lactam<br>Fluoroquinolone<br>Tetracycline<br>Sulfonamide<br>Trimethoprim	0.82	0.43	0.83
**Genotypic resistance vs. phenotypic resistance in *E. faecalis***				
**Period**	**AM classes**	**Procrustes SS**	**Correlation *r***	**Significance**
2001 - 2004	Amphenicol<br>Macrolide<br>Tetracycline	0.75	0.50	1

Significance of Procrustes rotation analysis between the PCoAs of annual estimates of phenotypic resistance in indicator E. coli, of genotypic resistance by metagenomics and of antimicrobial use (AMU). AM classes – antimicrobial classes included in the analysis; Procrustes *ss* - sum of squares represents the sum of squared differences between the two rotated PCoAs; Correlation *r*- correlation-like statistic (*r* = sqrt(1-*ss*)) that describes randomness between the two rotated PCoAs; Significance – significance of *r*. * P <0.5

Multivariate analysis of variance

Supplementary table S6 – Multivariate analysis of variance (MANOVA) for the difference in mean relative abundance of predicted resistance phenotypes between the sampling periods for metagenomics analysis 1999-2004 and 2015-2018.

**AMR class**	***Predicted phenotypic AMR w/significantly different alr means (*P* adj.<.05)**	**¥*N* ARGs**	**MANOVA**<br>**#*N* ARGs w/significantly different alr means (*P adj.* <.05)**	**ǂARGs w/significantly different alr means**	**†*P*-value (multivariate Pillai test)**
Aminoglycoside	Amikacin	5	2	aph.2__..Ib_2_AF207840<br>aac.6_..Im_1_AF337947	<.001
	Gentamicin	5	4	ant.2__..Ia_13_DQ176450<br>aph.2__..Ib_2_AF207840<br>aac.3..VIa_1_M88012<br>aac.3..VIa_2_NC_009838	0.03
	Streptomycin	36	8	ant.6..Ia_2_KF421157<br>ant.6..Ia_3_KF864551<br>ant.6..Ib_1_FN594949<br>aph.3__..Ib_5_AF321551<br>aadA13_2_NC010643<br>aadA14_1_AJ884726<br>aadA8b_1_AY139603<br>aadA8b_2_AM040708	<.001
	Tobramycin	5	3	ant.2__..Ia_13_DQ176450<br>aph.2__..Ib_2_AF207840<br>aac.6_..Im_1_AF337947	0.02
Beta-lactam	Ceftazidime	6	5	blaOXA.14_1_KJ420612<br>blaOXA.161_1_GQ202693<br>blaOXA.2_1_DQ112222<br>blaOXA.2_2_GQ466184<br>blaOXA.226_1_FJ617207	0.02

AMR class	*Predicted phenotypic AMR w/significantly different alr means (*P* adj.<.05)	¥*N* ARGs	MANOVA		
			#*N* ARGs w/significantly different alr means (*P adj.* <.05)	ǂARGs w/significantly different alr means	†*P*-value (multivariate Pillai test)
	Cephalothin	7	6	blaTEM.141_1_AY956335<br>blaTEM.1A_1_HM749966<br>blaTEM.1B_1_AY458016<br>blaTEM.1C_1_FJ560503<br>blaTEM.55_1_DQ286729<br>blaTEM.57_1_FJ405211	0.001
	Meropenem/ Imipenem	1	1	blaOXA.97_1_EF102240	0.02 (univariate F test)
	Piperacillin	18	5	blaCARB.11_1_AY008290<br>blaCARB.8_1_AY178993<br>blaOXA.10_1_J03427<br>blaTEM.1C_1_FJ560503<br>blaTEM.55_1_DQ286729	<.001
	Piperacillin-Tazobactam	3	3	blaOXA.10_1_J03427<br>blaOXA.14_1_KJ420612<br>blaTEM.40_1_FR717535	0.01
	Ticarcillin	9	7	blaTEM.141_1_AY956335<br>blaTEM.1A_1_HM749966<br>blaTEM.1B_1_AY458016<br>blaTEM.1C_1_FJ560503<br>blaTEM.40_1_FR717535<br>blaTEM.55_1_DQ286729<br>blaTEM.57_1_FJ405211	0.003
	Ticarcillin-Clavulanic acid	1	1	blaTEM.40_1_FR717535	0.01 (univariate F test)
Fosfomycin	Fosfomycin	2	1	fosA_3_ACWO01000079	0.01

AMR class	*Predicted phenotypic AMR w/significantly different alr means (**P** adj.<.05)	¥*N* ARGs	MANOVA		
			#*N* ARGs w/significantly different alr means (*P adj.* <.05)	ǂARGs w/significantly different alr means	†*P*-value (multivariate Pillai test)
Glycopeptide	Vancomycin	3	3	VanG2XY_1_FJ872410<br>VanGXY_1_AY271782<br>VanHBX_1_AF192329	<.001
Macrolide-Lincosamide-Streptogramin	Clindamycin (macrolide resistance genes)	23	6	erm.B._6_AF242872<br>erm.F._3_M17808<br>erm.G._1_M15332<br>erm.G._2_L42817<br>erm.Q._1_L22689<br>erm.X._4_NC_005206	<.001
	Erythromycin	31	9	erm.B._6_AF242872<br>erm.F._3_M17808<br>erm.G._1_M15332<br>erm.Q._1_L22689<br>erm.X._4_NC_005206<br>mef.A._2_U83667<br>mph.N._1_KF648874<br>msr.D._2_AF274302<br>msr.D._3_AF227520	<.001
	Pristinamycin IA; Quinupristin; Virginiamycin S.	22	9	erm.B._6_AF242872<br>erm.B._7_AF368302<br>erm.F._3_M17808<br>erm.G._1_M15332<br>[5] erm.G._2_L42817<br>erm.Q._1_L22689<br>erm.X._4_NC_005206<br>msr.D._2_AF274302<br>[9] msr.D._3_AF227520	<.001
	Spiramycin	2	2	mph.A._1_D16251<br>mph.B._1_D85892	0.006

AMR class	*Predicted phenotypic AMR w/significantly different alr means (*P* adj.<.05)	¥*N* ARGs	MANOVA		
			#*N* ARGs w/significantly different alr means (*P adj.* <.05)	‡ARGs w/significantly different alr means	†*P*-value (multivariate Pillai test)
	Telithromycin	4	4	mph.A._1_D16251<br>mph.B._1_D85892<br>msr.D._2_AF274302<br>msr.D._3_AF227520	<.001
	Azithromycin	8	8	mef.A._1_AJ971089<br>mef.A._2_U83667<br>mef.A._3_AF227520<br>mef.A._4_HG423652<br>mef.B._1_FJ196385<br>mph.A._1_D16251<br>msr.D._2_AF274302<br>msr.D._3_AF227520	<.001
Phenicol	Chloramphenicol	8	3	catQ_1_M55620<br>cfr.C._2_CANB01000378<br>floR_1_AF071555	<.001
	Florfenicol	4	4	cfr.C._1_KX686749<br>cfr.C._2_CANB01000378<br>floR_1_AF071555<br>floR_2_AF118107	<.001
Quinolone	Nalidixic acid	1	1	oqxB_1_EU370913	0.01 (univariate F test)
Sulfonamide	Sulfamethoxazole	20	3	sul1_18_AY260546<br>sul1_20_JF262165<br>sul2_2_AY034138	0.001

AMR class	*Predicted phenotypic AMR w/significantly different alr means (*P* adj.<.05)	¥*N* ARGs	MANOVA		
			#*N* ARGs w/significantly different alr means (*P adj.* <.05)	ǂARGs w/significantly different alr means	†*P*-value (multivariate Pillai test)
Tetracycline	Doxycycline; Tetracycline	67	26	tet.40._1_FJ158002<br>tet.40._2_AM419751<br>tet.44._1_NZ_ABDU01000081<br>tet.44._2_FN594949<br>tet.O._1_M18896<br>tet.O._2_M20925<br>tet.O._3_Y07780<br>tet.O.32.O._4_AIOQ01000025<br>tet.O.32.O._5_FP929050<br>tet.O.W..2_1_AY485122<br>tet.O.W._3_AM889120<br>tet.O.W._4_AM889121<br>tet.O.W._5_AM889122<br>tet.Q._1_L33696<br>tet.Q._2_X58717<br>tet.Q._3_U73497<br>tet.Q._4_Z21523<br>tet.W._2_AY049983<br>tet.W._4_FN396364<br>tet.W._5_AJ427422<br>tet.W.32.O._1_AM710601<br>tet.X._3_AB097942<br>tet.Y._1_EF495198<br>tetA.P._1_AB054980<br>tetA.P._2_L20800<br>tetB.P._1_NC_010937	<.001

AMR class	*Predicted phenotypic AMR w/significantly different alr means (*P* adj.<.05)	¥*N* ARGs	MANOVA		
			#*N* ARGs w/significantly different alr means (*P adj.* <.05)	ǂARGs w/significantly different alr means	†*P*-value (multivariate Pillai test)
	Minocycline	49	21	tet.44._1_NZ_ABDU01000081	<.001
				tet.44._2_FN594949	
				tet.O._1_M18896	
				tet.O._2_M20925	
				tet.O._3_Y07780	
				tet.O.32.O._4_AIOQ01000025	
				tet.O.32.O._5_FP929050	
				tet.O.W..2_1_AY485122	
				tet.O.W._3_AM889120	
				tet.O.W._4_AM889121	
				tet.O.W._5_AM889122	
				tet.Q._1_L33696	
				tet.Q._2_X58717	
				tet.Q._3_U73497	
				tet.Q._4_Z21523	
				tet.W._2_AY049983	
				tet.W._4_FN396364	
				tet.W._5_AJ427422	
				tet.W.32.O._1_AM710601	
				tet.X._3_AB097942	
				tetB.P._1_NC_010937	
Trimethoprim	Trimethoprim	19	11	dfrA1_10_AF203818	0.07
				dfrA1_11_AJ419168	
				dfrA1_13_DQ018382	
				dfrA1_17_FJ489928	
				dfrA14_1_KF921535	
				dfrA14_3_Z50804	
				dfrA14_4_AF393510	
				dfrA14_5_DQ388123	

**AMR class**	***Predicted phenotypic AMR w/significantly different alr means (*P* adj.<.05)**	**¥*N* ARGs**	**MANOVA**		
			**#*N* ARGs w/significantly different alr means (*P adj.* <.05)**	**‡ARGs w/significantly different alr means**	**†*P*-value (multivariate Pillai test)**
				dfrA5_1_X12868<br>dfrA5_2_FJ001870<br>dfrA8_1_U10186	

MANOVA to assess significant changes in mean relative abundance (alr) of individual resistance genes between the periods 1999-2004 and 2015-2018, for those phenotypes that have shown a significant difference between those periods.*Resistance phenotypes (predicted) with significant (P <.05) difference in alr means between the periods 1999-2004 and 2015-2018; ¥number of individual antimicrobial resistance genes (ARG) annotated to a specific phenotype, among all hit ARGs; #number of individual ARGs w/significantly different alr means between the periods 1999-2004 and 2015-2018 (*P*<.05 in univariate F-test, adjusted by Holm method); ‡individual ARGs w/significantly different alr means between the periods 1999-2004 and 2015-2018 (individual genes are in the Resfinder database named by their scientific name, variant and genebank accession number); †*P*-values for multivariate Pillai test for the difference of phenotype-alr means between the periods 1999-2004 and 2015-2018, considering all genes annotated to a phenotype (*N* ARGs).

Supplementary figure S1- Abundance of individual resistance genes within an antimicrobial class by sampling year

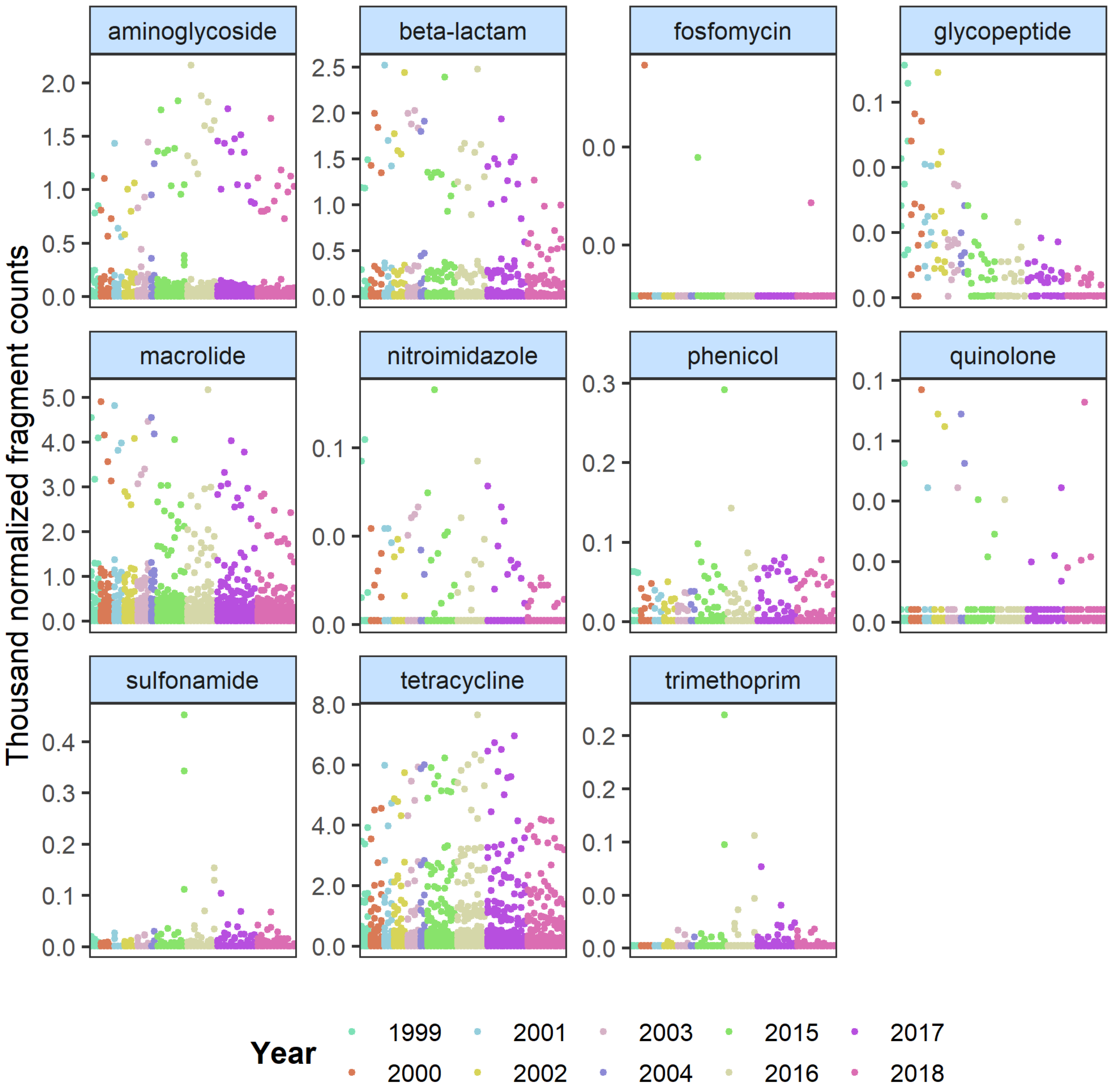

Each point within a given year represents an individual ARG of the corresponding antimicrobial class.

Supplementary figure S2 - Comparison of phenotypic resistance in indicator *E. coli* with relative abundance of resistance in resistomes.

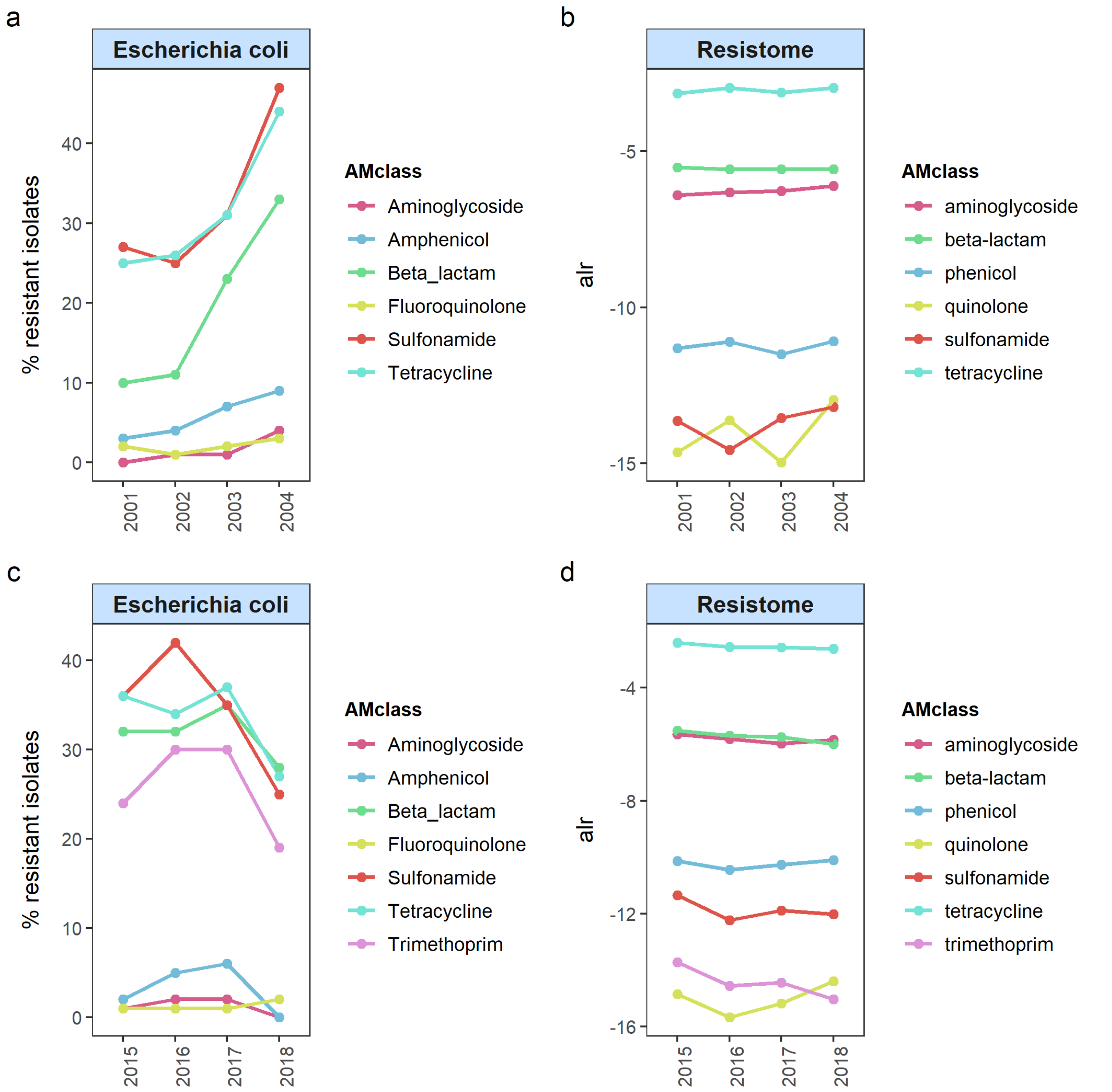

Annual percentage of indicator *E. coli* isolates phenotypically resistant to different antimicrobial classes, in the sampling period 2001-2004 (a) and 2015-2018 (c) ; Relative abundance of matching antimicrobial class resistance (alr-transformed ARG counts), calculated for counts aggregated at year level, for sampling period 2001-2004 (b) and 2015-2018 (d).

Resistome clustering and ordination analysis

Supplementary figure S3 – Clustering of resistomes aggregated at antimicrobial resistance gene level

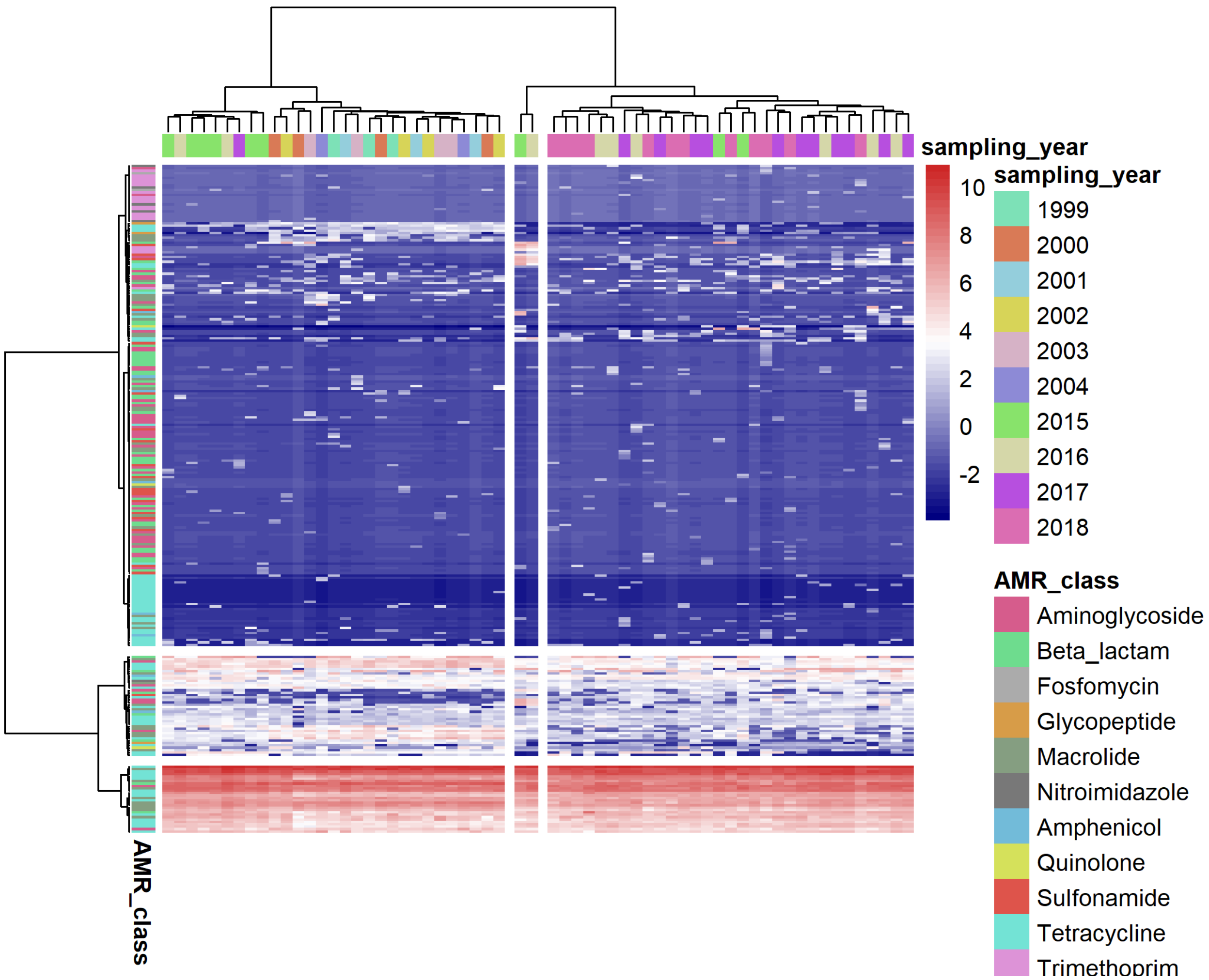

Centered log ratio (clr) transformed count matrices were used to produce a heatmap at antimicrobial resistance gene level (*a*) The value for each ARG in a sample represents that gene's ratio to the average abundance across all genes in the same sample. Samples were clustered by row (antimicrobial class) and by column (year) based on Euclidean distances and by Ward agglomeration method (dissimilarities not squared). The continuous color scale represents clr values, from red (high) to blue (low).

Supplementary figure S4 - Multivariate analysis of variance (MANOVA) for the difference in mean relative abundance of predicted resistance phenotypes between the two sampling periods 1999-2004 and 2015-2018.

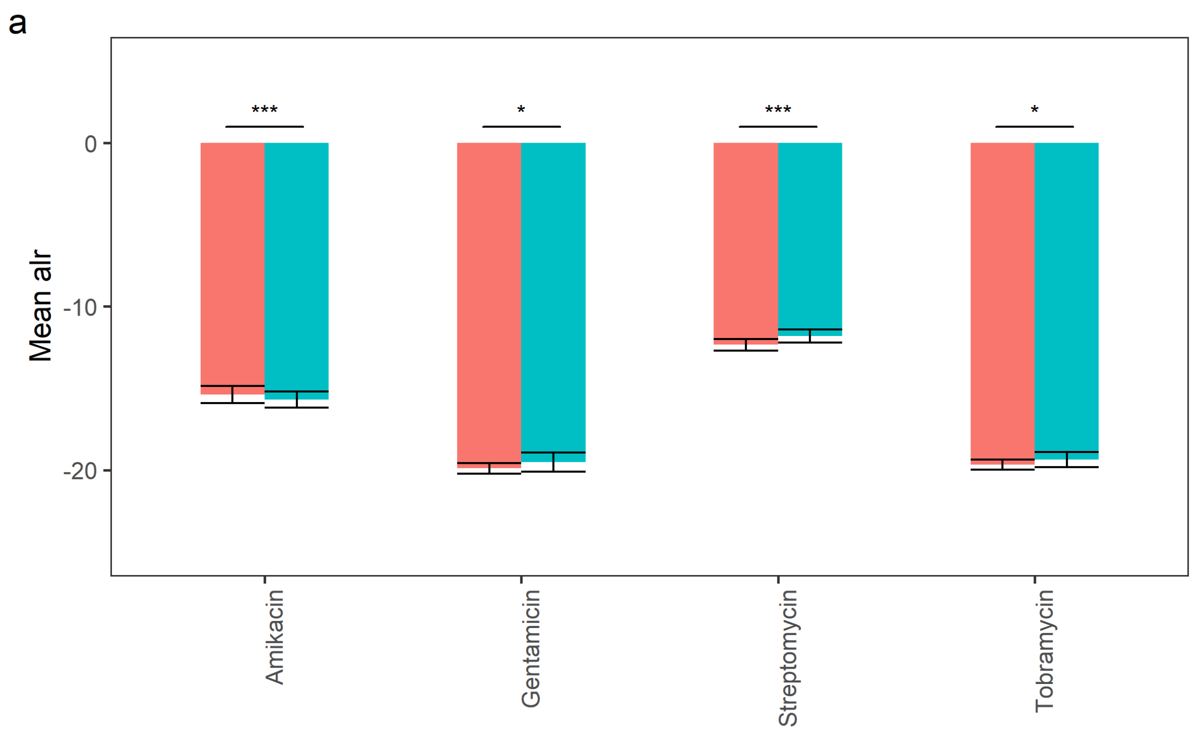

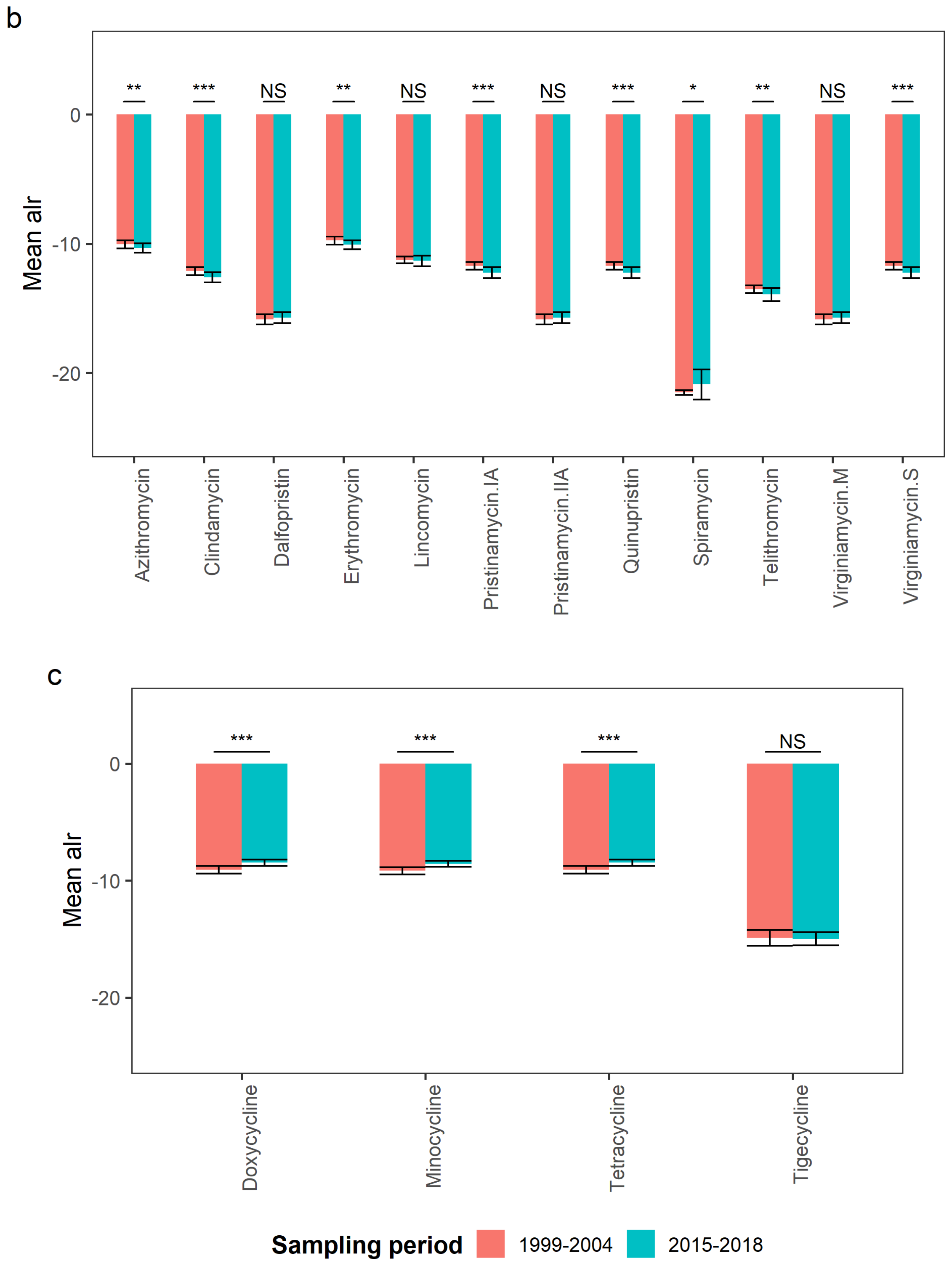

Mean relative abundance in the resistome (alr) of all genes annotated to individual predicted resistance phenotypes, measured in two sampling periods, for the antimicrobial classes aminoglycoside (*a*), macrolide-lincosamide-streptogramin (*b*), and tetracycline (*c*). The error bars represent standard deviation. The symbols represent the

statistical level of significance of the difference in mean alr between the two sampling periods (*NS*: nonsignificant ($P > 0.05$), *$P \leq 0.05$, **$P \leq 0.01$, ***$P \leq 0.001$).

Supplementary figure S5 – Time-series cross-correlation significant coefficients for increased AMU leading to increased AMR measured by shotgun metagenomics and as phenotypic resistance in indicator *E. coli*.

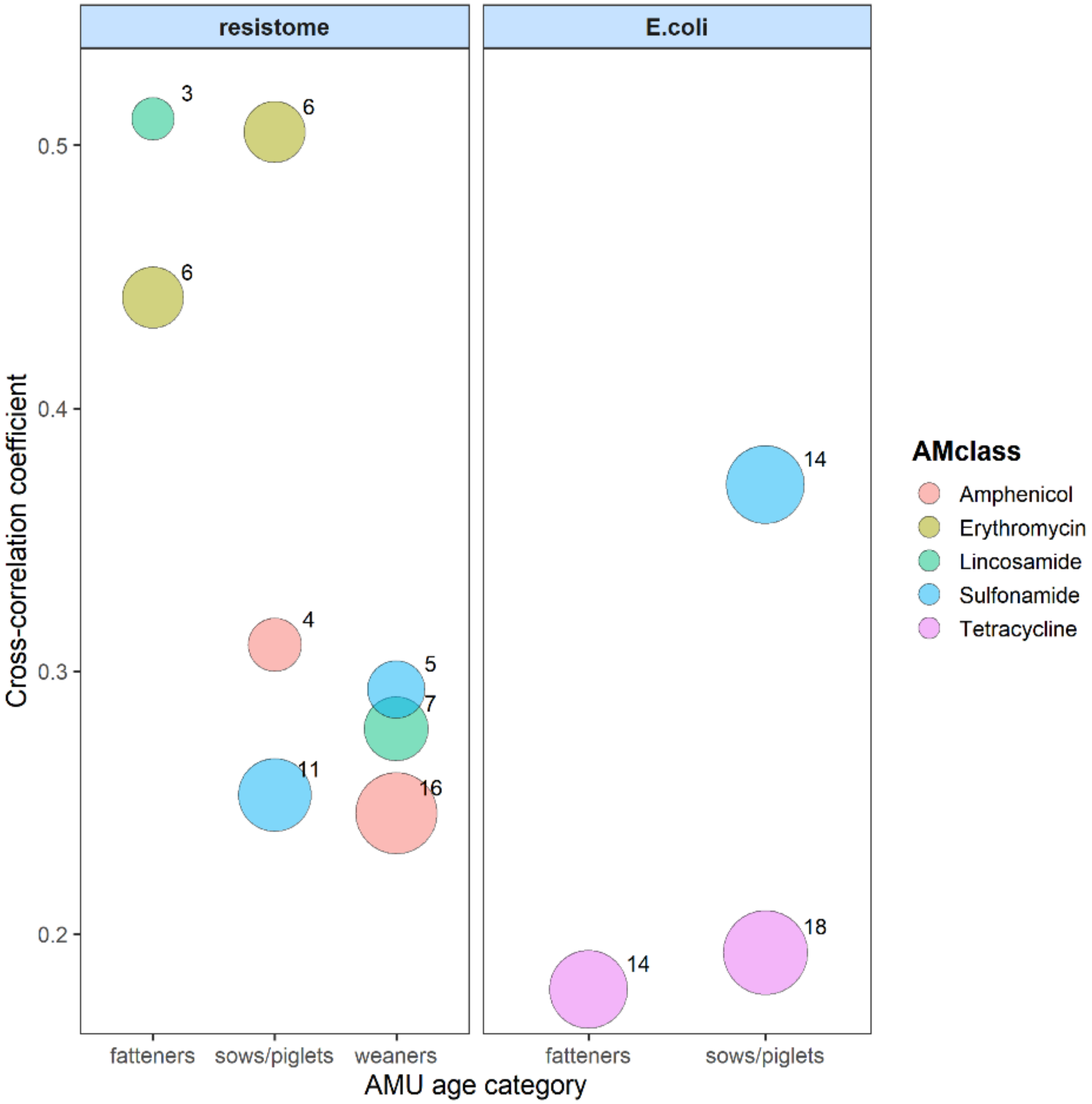

Highest significant cross-correlation coefficients among positive coefficients with negative lag (supplementary tables 5 and 6). Results are shown for three AMU groups – sows/piglets, weaners and fatteners (*x-axis*) - and two measures of AMR in finisher pigs at slaughter – relative abundance in resistome and percentage of resistant *E. coli* isolates. Cross-correlation coefficients are shown in the *y-axis*. The bubble size relates to the magnitude of the lag between increased AMU and increased AMR, which is also shown as the number of lag months on the top-right

corner of each bubble.. These results represent scenarios where higher AMU in a specific age group leads to higher AMR measured in finishers at slaughter.

Supplementary methods

Time-series cross-correlation analysis

Cross-correlation functions depend on the assumptions that there is no autocorrelation and that series are stationary, which are verified by visually inspecting the components of each time-series, and correcting them accordingly. Time-series stationarity is frequently obtained by differencing the data[1], with one step differencing being usually sufficient to detrend the time-series. After detrending, the seasonality becomes clearer. Here, removal of the time-series seasonality component was done after detrending, using the *seasadj* function of R time-series *forecast* package[2]. At each moment of differentiation and seasonal component removal, we inspected for stationarity by performing the non-parametric test for stationarity Kwiatkowski-Phillips-Schmidt-Shin test (KPSS) and the augmented Dickey–Fuller test (ADF). We selected the series with the least amount of differencing. Missing data was interpolated using linear interpolation[3]. All time-series were log transformed, and standardized by z-transformation using R package *dtwclust*[4,5] to facilitate comparison of cross-correlations.

To determine if one time series had an effect on another, whether instantaneously or at a different point in time, we looked at cross-correlation coefficient plots and tested the significance of pairwise correlations. It is standard practice to reject the hypothesis that the population cross-correlation of lag k equals zero for a significance level of approximately 5%. Here we chose robust methods for estimating significance of cross-correlations[6], using the R package *testcorr*[7]. Figure 10 shows an example of a cross correlation plot where standard (assuming approximation to normal distribution) and the here applied robust thresholds are represented, and the interpretation of results is illustrated.

Supplementary figure S6: An example of a cross-correlation plot for interpreting coefficients at different lags.

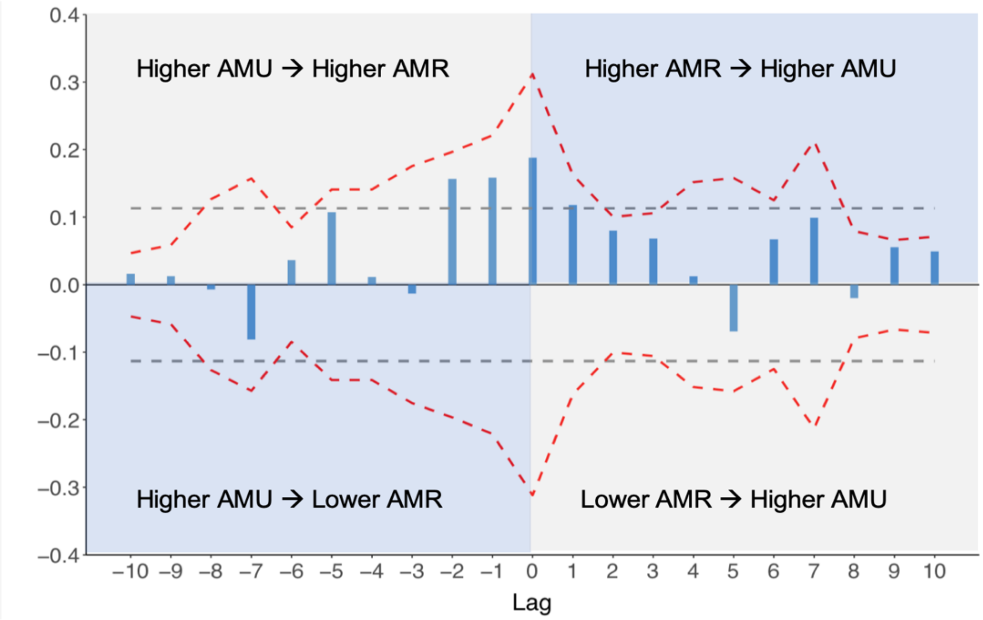

Straight, grey lines define thresholds for standard cross-correlation significance, while irregular, red lines define the threshold of significance according to the robust methodology by Dalla et al. (2020a). Here, we consider significant cross-correlations those above (top) or below (bottom) the red lines estimated using the robust methodology. The interpretation given to significant cross-correlations is indicated in each quadrant. Cross-correlation coefficients at significant negative lags can be interpreted as higher than average AMU leading to a higher or lower AMR, for positive and negative cross-correlation coefficients, respectively. In turn, positive lags reflect higher AMR leading to higher or lower AMU, for positive and negative coefficients, respectively. Any association is such that it occurs at the number of months indicated by the lag value later in the future. The value of the lag with the highest correlation coefficient represents the best fit between two series.

The above described cross-correlation estimation method will frequently overestimate associations due to strong patterns in the structure of the individual time-series, as is the case of auto-correlation. For that reason, we accounted for the univariate ARIMA (AutoRegressive Integrated Moving Average) structure of AMU time-series, before determining cross-correlation with resistance, following the steps by Probst et al., 2012. First we decided on autoregressive (AR)/ moving average (MA) orders to achieve the best ARIMA model for the AMU time series

according to AIC value, through the auto.arima function in the forecast R package; second, we checked the auto correlation function (ACF) plot of residuals of the transformed AMU series (Hyndman et al., 2020); third, we applied the same transformation to the AMR time-series to preserve equality, using R stats and forecast package functions[2]; last, we performed the cross-correlation on the residuals considering robust thresholds.

Supplementary table S7 - Time-series cross-correlation coefficients for monthly measurements of antimicrobial resistance by metagenomics in finisher pigs at slaughter against monthly measurements of antimicrobial use in sows/piglets, weaners and fatteners in the period 2015-2018.

**AMR class/substance**	**Sows/Piglets**		**Weaners**		**Fatteners**	
	**Positive**	**Negative**	**Positive**	**Negative**	**Positive**	**Negative**
Tetracycline	-	-	0.313 (10)<br>0.256 (4)	-0.347 (-18)<br>-0.292 (-14)<br>-0.302 (7)	0.378 (6)	-0.309 (-18)
Erythromycin	0.505 (-6)<br>0.431 (-1)	-0.185 (-19)<br>-0.273 (18)	-	-0.316 (13)<br>-0.288 (14)<br>-0.302 (18)	0.442 (-6)<br>0.339 (0)	-
Sulfonamide	0.253 (-11)<br>0.250 (-10)<br>0.317 (1)	0.263 (-6)<br>-0.499 (4)<br>-0.213 (13)	0.293 (-5)<br>0.419 (0)<br>0.346 (3)<br>0.464 (6)	-0.515 (-1)<br>-0.396 (2)<br>-0.353 (4)	-	-
Amphenicol	0.310 (-4)	-0.238 (-5)<br>-0.144 (20)	0.246 (-16)	-0.387 (-3)	0.245 (20)	-0.378 (-4)
Lincosamide	0.316 (7)	-0.288 (19)	0.278 (-7)<br>0.359 (12)	-0.450 (-4)	0.510 (-3)	-0.360 (9)<br>-0.333 (15)
Neomycin	-	-	0.345 (0)<br>0.257 (10)	-0.323 (-1)	-	-

The cross-correlation coefficients between trends in AMU at the farm and genotypic AMR at slaughter in the period 2015-2018 were analysed for several antimicrobials/antimicrobial classes (depending on data availability). Results are shown considering AMU in three groups – sows/piglets, weaners and fatteners, including cross-correlation coefficients and lags in months. Cross-correlation negative lags represent scenarios where AMU leads AMR, whereas positive lags represent AMR leading AMU. Positive coefficients with negative lags are in grey highlight, indicating scenarios where increased AMU leads to increased AMR.

Supplementary table S8 - Time-series cross-correlation coefficients for monthly measurements of antimicrobial resistance in indicator *E. coli* isolated from finisher pigs at slaughter against monthly measurements of antimicrobial use in sows/piglets, weaners and fatteners in the period 2015-2018.

**AMR class/substance**	**Sows/Piglets**		**Weaners**		**Fatteners**	
	**Positive**	**Negative**	**Positive**	**Negative**	**Positive**	**Negative**
Tetracycline	0.193 (-18)<br>0.161 (-5)	-0.508 (0)<br>-0.214 (6)	0.370 (9)	-	0.179 (-14)	(-0.367 (0)<br>-0.256 (-9)
Sulfonamide	0.371 (-14)	-0.252 (-17)<br>-0.250 (-9)<br>-0.394 (9)<br>-0.403 (10)	0.345 (4)<br>0.313 (12)	-0.235 (12)	-0.206 (11)	-
Amphenicol	-	-	0.323 (6)	-	-	-

The cross-correlation coefficients between trends in AMU at the farm and phenotypic AMR in indicator *E. coli* at slaughter in the period 2015-2018 were analysed for several antimicrobials/antimicrobial classes (depending on data availability). Results are shown considering AMU in three groups – sows/piglets, weaners and fatteners, including cross-correlation coefficients and lags in months. Cross-correlation negative lags represent scenarios where AMU leads AMR, whereas positive lags represent AMR leading AMU. Positive coefficients with negative lags are in grey highlight, indicating scenarios where increased AMU leads to increased AMR.

Extended data references

1. Hossain, Z., Rahman, A., Hossain M. & Karami J.H. (2019). Differencing and Forecasting with Non-Stationary Time Series Data. *Dhaka Univ. J. Sci.*, 67(1), 21-26.
2. Hyndman, R., et al. (2020). forecast: Forecasting functions for time series and linear models. http://pkg.robjhyndman.com/forecast.
3. Zeileis, A., & Grothendieck, G. (2005). zoo: S3 Infrastructure for Regular and Irregular Time Series. *Journal of Statistical Software*, 14(6), 1-27. https://doi.org/doi:10.18637/jss.v014.i06
4. Sarda-Espinosa A. (2019). dtwclust: Time Series Clustering Along with Optimizations for the Dynamic Time Warping Distance. https://CRAN.R-project.org/package=dtwclust
5. Blanchard, J.L., et al. (2010). Trend analysis of indicators: a comparison of recent changes in the status of marine ecosystems around the world. *ICES Journal of Marine Science*, 67(4), 732–744. https://doi.org/10.1093/icesjms/fsp282

6. Dalla, V., Giraitis, L., & Phillips, P. (2020a). Robust Tests for White Noise and Cross-Correlation. *Econometric Theory*, 1-29. https://doi.org/10.1017/S0266466620000341
7. Dalla, V., Giraitis. L., & Phillips, P. (2020b). testcorr: Testing Zero Correlation. https://CRAN.R-project.org/package=testcorr.
